# Skin perfusion pressure as an indicator of tissue perfusion in valvular heart surgery: Preliminary results from a prospective, observational study

**DOI:** 10.1371/journal.pone.0184555

**Published:** 2017-09-19

**Authors:** Young Song, Sarah Soh, Jae-Kwang Shim, Kyoung-Un Park, Young-Lan Kwak

**Affiliations:** 1 Department of Anesthesiology and Pain Medicine, Yonsei University College of Medicine, Seodaemun-gu, Seoul, Republic of Korea; 2 Anesthesia and Pain Research Institute, Yonsei University College of Medicine, Seodaemun-gu, Seoul, Republic of Korea; Universita degli Studi di Bologna, ITALY

## Abstract

Hemodynamic management aims to provide adequate tissue perfusion, which is often altered during cardiac surgery with cardiopulmonary bypass (CPB). We evaluated whether skin perfusion pressure (SPP) can be used for monitoring of adequacy of tissue perfusion in patients undergoing valvular heart surgery. Seventy-two patients undergoing valve replacement were enrolled. SPP and serum lactate level were assessed after anaesthesia induction (baseline), during CPB, after CPB-off, end of surgery, arrival at intensive care unit, and postoperative 6 h. Lactate was further measured until postoperative 48 h. Association of SPP with lactate and 30-day morbidity comprising myocardial infarction, acute kidney injury, stroke, prolonged intubation, sternal infection, reoperation, and mortality was assessed. Among the lactate levels, postoperative 6 h peak value was most closely linked to composite of 30-day morbidity. The SPP value during CPB and its % change from the baseline value were significantly associated with the postoperative 6 h peak lactate (r = -0.26, P = 0.030 and r = 0.47, P = 0.001, respectively). Optimal cut-off of % decrease in SPP during CPB from baseline value for the postoperative 6 h hyperlactatemia was 48% (area under curve, 0.808; 95% confidence interval (CI), 0.652–0.963; P = 0.001). Decrease in SPP >48% during CPB from baseline value was associated with a 12.8-fold increased risk of composite endpoint of 30-day morbidity (95% CI, 1.48–111.42; P = 0.021) on multivariate logistic regression. Large decrease in SPP during CPB predicts postoperative 6 h hyperlactatemia and 30-day morbidity, which implicates a promising role of SPP monitoring in the achievement of optimal perfusion during CPB.

## Introduction

Maintenance of adequate tissue oxygenation is a prerequisite for better prognosis in cardiac surgical patients, and thus, the highest priority in their perioperative management should be sufficient global tissue perfusion [1, 2]. Still, current hemodynamic resuscitation during cardiac surgery relies on parameters like blood pressures and cardiac output, whose relevance as indicators for optimal tissue perfusion in critically ill patients have been debated [3–5]. Hence, a growing need exists for precise measurement of microcirculation, which can be easily disrupted independent of systemic hemodynamics in cardiac surgical patients, especially during cardiopulmonary bypass (CPB) [6, 7].

For decades, blood lactate concentration has been the most commonly used surrogate marker of tissue perfusion bearing association with prognosis [8]. Its utility as a bedside monitoring tool in cardiac surgery, however, is questionable mainly because of the inability to reflect real-time perfusion status and cumbersome nature of the multiple blood sampling [9]. As yet, there is no laboratory test or monitor that allows reliable assessment of tissue perfusion with proven clinical feasibility [10].

Skin perfusion pressure (SPP) is a widely used noninvasive test for the quantitative analysis of microcirculation in peripheral arterial disease (PAD) patients [11]. It utilizes a laser Doppler sensor and a pressure cuff to measure the pressure at which blood flow first returns to capillaries following controlled occlusion and subsequent release [12]. Its strength lies on the ease in use, simplicity, and being readily interpretable, and more importantly, its accuracy hardly falls behind other technologies in determining peripheral perfusion [13–16]. However, its role as a monitoring tool in other clinical circumstances where microcirculatory disturbance may occur has never been evaluated.

Valvular heart surgery is at relatively constant risk of perioperative derangements of microcirculation resulting from pre-existing cardiovascular dysfunction and certain period of exposure on the CPB and cardiac ischemia [2, 17]. Also, these patients are less likely to be associated with undiagnosed PAD compared to those undergoing coronary or aortic surgery [18]. Hence, we aimed to investigate the role of perioperative SPP monitoring as an indicator of tissue perfusion assessed by serum lactate levels in patients receiving valvular heart surgery using CPB.

## Materials and methods

This study has been approved by Yonsei University Health System IRB (reference number 1-2014-0070) and informed consent was obtained from all the participants.

### Study participants

After approval by the institutional review board (reference number 1-2014-0070) and registration at ClinicalTrials.gov (NCT02329392), this prospective, observational study was conducted at Yonsei University Health System, Seoul, Republic of Korea. A total of 72 patients (≥20 years old) scheduled for elective valve surgery with cardiopulmonary bypass (CPB) were enrolled after they provided written informed consent. Exclusion criteria were known PAD, defined as angiographic narrowing of a lower limb artery, evidence of narrowing on ultrasound-duplex, or ankle-brachial index ≤0.90 [19], serum lactate level ≥2 mmol/L, surgeries planned without the use of pulmonary artery catheter, active liver disease or cirrhosis, end-stage renal disease, infective endocarditis, heart transplantation, a state of cardiogenic shock, or mechanical circulatory support.

### Study endpoints

The primary endpoint of this study was to evaluate the association of perioperative SPP values with serum lactate levels. The secondary endpoint was to assess the ability of perioperative SPP measurement as a predictor of hyperlactatemia and postoperative 30-day morbidity.

### Perioperative management

All patients received standardized perioperative care according to our institutional guidelines [20]. Perioperative monitoring employed the following: 20-G radial artery catheter, cerebral near infrared spectroscopy (NIRS;INVOS 4100,Covidien, Mansfield, MI), thermodilutional pulmonary artery catheter inserted via the internal jugular vein (Swan-Ganz CCOmbo CCO/SvO_2_TM, Edwards Lifesciences LLC, Irvine, CA), nasopharyngeal probes, urinary catheter, and skin temperature probes at the dorsal foot. Transesophageal echocardiography was used during the surgery in all patients. Anesthesia was maintained with sevoflurane and continuous infusion of sufentanil. All the surgery was performed via mid-sternotomy. CPB was primed with a 1.6 L solution consisting of 100 mL of 20% albumin, 0.5g/kg of 20% mannitol, 20 mEq of sodium bicarbonate, 2000 IU heparin, and acetated Ringer’s solution. The CPB was established with central cannulation through ascending aorta in all cases and was free from possible influence on the peripheral perfusion by femoral arterial cannulation. Non-pulsatile pump flow was maintained at a rate of 2.0–2.4 L/min/m^2^ under mild systemic hypothermia (32–33°C) with α-stat management. During aortic cross clamp (ACC), cold blood cardioplegia was used intermittently via the aortic root or directly through the coronary ostia. After the main surgical procedure, patients were rewarmed to urinary catheter temperature of >35.5°C, followed by weaning from CPB. After sternal closure, all patients were transferred to the intensive care unit (ICU).

To maintain the mean arterial pressure (MAP) between 60 and 80 mmHg, norepinephrine was primarily titrated to a maximum of 0.3μg/kg/min and vasopressin up to 4 IU/h was added. Milrinone was administered when the cardiac index (CI) was below 2L/min/m^2^ despite an adequate volume status. The hematocrit level was maintained above 20% during CPB and 25% before and after CPB with the use of ultrafiltration during CPB as well as transfusion of packed red blood cells (pRBC) and reinfusion of the intraoperatively salvaged blood.

### Study protocol

SPP was measured at the dorsum of both foot using SensiLase PAD-IQ (Vasamed Inc., Eden Prairie, MN, USA). The machine had a laser Doppler sensor attached over the center of the dorsum and a blood pressure cuff that wrapped around both the sensor and the foot. The sensor identified perfusion as the presence of a Doppler frequency shift generated by the motion of RBCs of the capillary flow in 1.5-mm skin depth. The cuff automatically inflated as determined by the sensor to occlude skin perfusion for 10 s, followed by release at a rate of 10 mmHg/5 s. Changes in cuff pressure versus % perfusion and the SPP value, which is defined as the pressure at which moving RBCs were first detected following the cuff release, were displayed graphically on each measurement. The mean of the values of both feet was used in the final analysis.

Hemodynamic parameters, including MAP, CI, central venous pressure (CVP), pulmonary arterial diastolic pressure (PADP), mixed venous oxygen saturation (SvO_2_), core (nasopharyngeal)-to-peripheral (skin) temperature gradient (CPTG), cerebral regional oxygen saturation (CRSO_2_), and SPP were measured at various predetermined time points as follows: after induction of anesthesia (baseline), during hypothermic CPB repeated at 30-min intervals, immediately after weaning from CPB, after sternal closure, after arrival at the ICU, and postoperative 6 h (average of 2, 4, and 6 h).

Serum lactate levels obtained from arterial blood sampling were measured at the same time points as above and at postoperative 12, 24, and 48 h. Subsequently, the mean and peak levels during the surgery and postoperative 6 h (0–6 h), 12 h (0–12 h), 24 h (0–24 h), and 48 h (0–48 h) were determined.

Postoperative 30-day morbidity endpoints, including new-onset myocardial infarction (defined as an increase in CK-MB or Tn-T levels over the 99^th^ percentile upper reference limit, in case of a normal preoperative value, or over five times the upper normal limit with a newly developed Q wave or left bundle branch block, in case of an abnormal preoperative value), acute kidney injury (AKI; defined as an increase in serum creatinine >0.3 mg/dL or 50% from baseline) [21], stroke, mechanical ventilation >24 h, deep sternal wound infection, reoperation, mortality, and composite of the above.

### Other assessments

Preoperative data collection included demographic details; history of hypertension, diabetes, coronary artery disease, chronic kidney disease (defined as serum creatinine level ≥2mg/dL), and chronic obstructive pulmonary disease, and previous cardiac surgery; EuroSCORE; left ventricular ejection fraction; New York Heart Association (NYHA) functional classification; cardiovascular medication; and serum hemoglobin, creatinine, and lactate levels. Intraoperative data collection included type of surgery, CPB time, ACC time, pRBC transfusion, amount of ultrafiltration, and requirement of vasopressor and inotropic agents. Postoperative data collection included requirement of vasopressor and inotropic agents, and pRBC transfusion for the first 6 h.

### Statistical analysis

Sample size calculation was performed using PASS (NCSS Statistical Software, 2013). From our institution’s previous database, we obtained a correlation coefficient (r) of -0.20 (P = 0.128) between the average of CRSO_2_ values during surgery and postoperative 48 h peak lactate level in 200 adult patients undergoing valvular heart surgery. In a recent clinical study [22], perioperative thenar muscle tissue oxygen saturation (StO_2_), a widely used indicator of microcirculation, was significantly correlated with serum lactate clearance (r = 0.46, P <0.01). Assuming a similar significant association between perioperative SPP value and lactate level (r = 0.45), we determined that 66 subjects would be required to detect a difference of 0.25 between the correlation coefficients of CRSO_2_-lactate and SPP-lactate with an 80% power and type I error of 5%. Considering a possible 10% dropout rate, we decided to enroll 72 patients.

Perioperative SPP values and their percent changes from the baseline value are shown as median [interquartile range]. Associations of the hemodynamic parameters and their percent changes from the baseline value with serum lactate level were determined by Pearson correlation analysis. For the comparison of continuous variables, independent *t*-test or Mann-Whitney *U* test was used. Categorical variables were compared using the chi-square or Fisher’s exact test, as appropriate. Discriminative capacity of percent change of SPP from baseline and its cutoff value providing the greatest sum of sensitivity and specificity to detect hyperlactatemia was assessed using a receiver operating characteristic (ROC) curve. To calculate odds ratios (OR) and 95% confidence intervals (95% CI) for the determination of the independent predictive power of the percent change in SPP for the 30-day morbidity, a logistic regression analysis was performed. Variables with P value <0.1 between patients with and those without a composite endpoint of 30-day morbidity were first evaluated in the univariate model. Variables with a P value <0.05 were entered into the multivariate model. Statistical analysis was performed using SPSS version 23.0 (IBM Corp., Armonk, NY, USA). A P value <0.05 was considered statistically significant.

## Results

**Fig 1:**
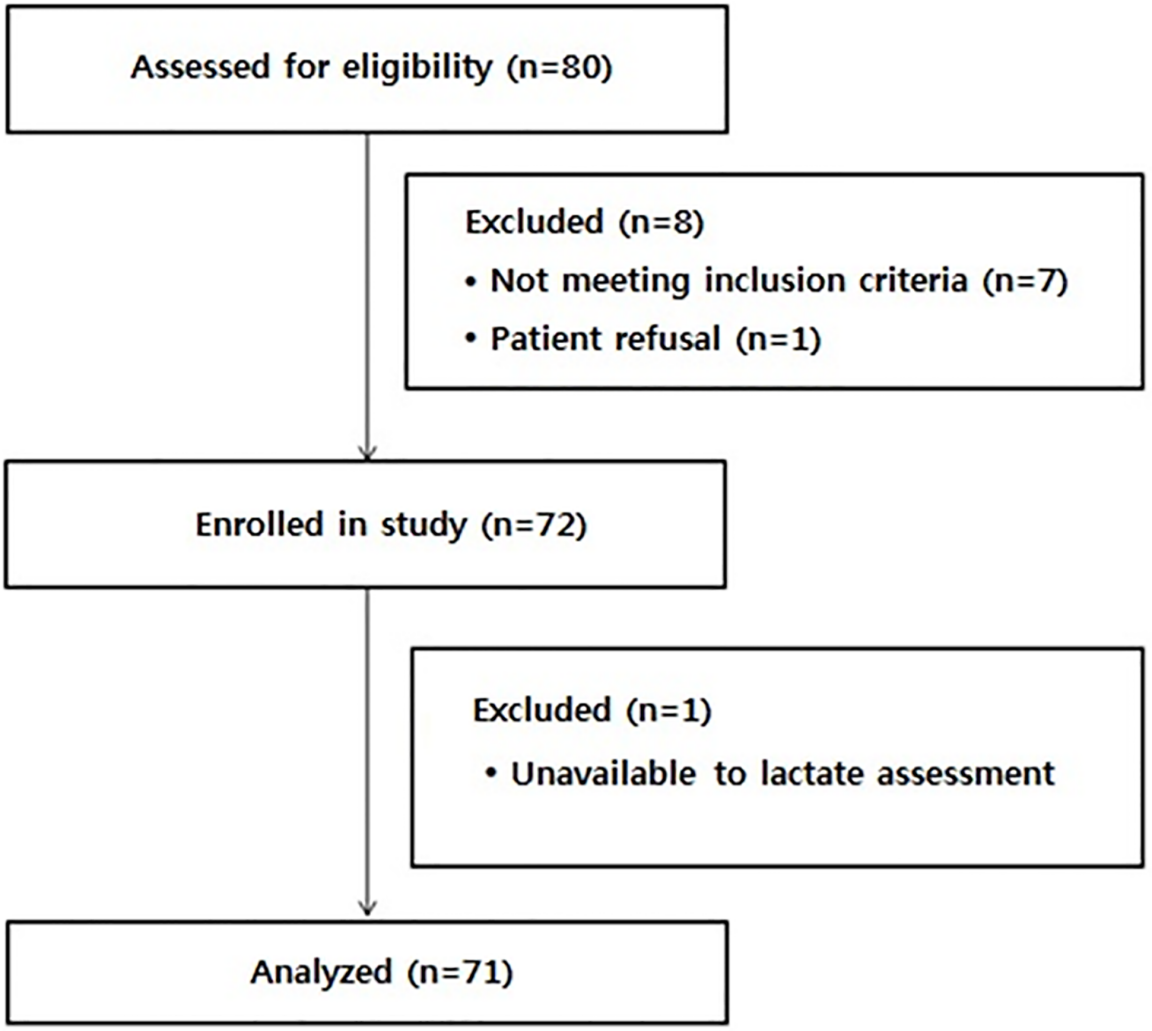
Flow diagram of study participants.

Among the 72 enrolled patients, one subject was excluded because of missing lactate levels due to measurement error. Thus, a total of 71 patients were included in the final analysis (Fig 1).

### Relationship between SPP and serum lactate level

Among the lactate levels assessed, the peak value of postoperative 6 h yielded an area under the ROC curve (AUC) of the best discriminative capacity in patients with and without a composite endpoint of 30-day morbidity (AUC, 0.762; 95% CI, 0.602–0.922, P = 0.004, S1 Fig). The optimal cutoff value for the prediction was 3.0 mmol/L with a sensitivity and specificity of 66.7 and 84.7%, respectively.

Among SPP values of 6 time points, the value during CPB was negatively associated with the postoperative 6 h peak lactate (r = -0.26, P = 0.030). Percent changes of SPP values during CPB and at the end of surgery from the baseline value were also significantly correlated to the postoperative 6 h peak lactate (r = -0.47, P = 0.001 and r = -0.31, P = 0.045, respectively) (Table 1).

**Table 1:** Perioperative SPP values and their associations with the postoperative 6 h peak lactate level.

	SPP, mmHg	r	Change of SPP from baseline, %	r
Post-IND	56 [36−78]	0.08		0.01
During CPB	40 [33−54]	-0.26*	-42 [-54−-21]	0.47 †
After CPB-off	51 [31−67]	-0.13	-14 [-45−12]	0.20
Op end	65 [39−91]	-0.16	-22 [-49−-5]	0.31*
After ICU arrival	52 [37−71]	-0.163	-24 [-53−-1]	0.20
Postoperative 6 h	49 [34−74]	-0.09	-9 [-37−44]	0.07

The postoperative 6 h peak lactate level was 2.5 [2.0–3.0] mmol/L. SPP values and their percent changes are expressed as median [IQR]. Change of SPP was calculated as a difference between the measured value and baseline (Post-IND) value divided by baseline value X 100 (%). r = correlation coefficient between each SPP value (and % change from the baseline value) and postoperative 6 h peak lactate assessed by Pearson correlation analysis. SPP = skin perfusion pressure; Post-IND = after induction of anesthesia; CPB = cardiopulmonary bypass; ICU = intensive care unit.

* = P < 0.05;

^†^ = P <0.01

Associations of conventional hemodynamic parameters (and % changes from the baseline value) with postoperative 6 h peak lactate level are shown in Table 2. The MAP after induction of anesthesia (r = 0.29, P = 0.013), CRSO_2_ values after weaning from CPB (r = -0.27, P = 0.021) and at the end of surgery (r = -0.24, P = 0.045) were significantly correlated with lactate level. Percent changes of CI at ICU arrival and MAP at postoperative 6 h from the baseline values were also significantly correlated with the postoperative 6 h peak lactate (r = -0.24, P = 0.045 and r = -0.28, P = 0.022, respectively)

**Table 2:** Relationship between hemodynamic parameters and postoperative 6 h peak lactate level.

		Absolute value						
		MAP	CI	CVP	PADP	SvO2	CPTG	CRSO2
Post-IND	r	0.29*	0.20	0.03	-0.09	-0.04	0.21	-0.17
During CPB	r	0.03	0.03				0.36	-0.10
After CPB-off	r	0.07	0.02	-0.01	-0.07	-0.23	0.07	-0.27*
Op end	r	-0.07	-0.14	0.09	-0.09	-0.08	0.28	-0.24*
After ICU arrival	r	0.02	-0.04	-0.04	-0.10	-0.22	-0.01	0.06
Postoperative 6 h	r	-0.20	-0.11	-0.12	-0.03	-0.19	0.39	-0.20
		% change from the baseline value						
		MAP	CI	CVP	PADP	SvO2	CPTG	CRSO2
During CPB	r	-0.23	0.13				-0.06	-0.15
After CPB-off	r	-0.19	-0.07	-0.02	0.05	-0.04	0.05	0.22
Op end	r	-0.13	-0.20	0.01	0.10	0.06	0.12	0.13
After ICU arrival	r	-0.22	-0.28*	-0.06	-0.01	-0.02	-0.13	0.02
Postoperative 6 h	r	-0.24*	-0.22	-0.01	-0.09	0.04	-0.14	-0.25

The postoperative 6 h peak lactate level was 2.5 [2.0–3.0] mmol/L. r = correlation coefficient between each hemodynamic variable (and % change from the baseline value) and postoperative 6 h peak lactate assessed by Pearson correlation analysis. Post-IND = after induction of anesthesia; CPB = cardiopulmonary bypass; ICU = intensive care unit; MAP = mean arterial pressure; CI = cardiac index (pump flow rate during CPB); CVP = central venous pressure; PADP = pulmonary arterial diastolic pressure; SvO_2_ = mixed venous oxygen saturation; CPTG = Core to peripheral (skin) temperature gradient; CRSO_2_ = cerebral regional oxygen saturation.

* = P < 0.05.

### Optimal cutoff of the % change in SPP during CPB to detect hyperlactatemia

**Fig 2:**
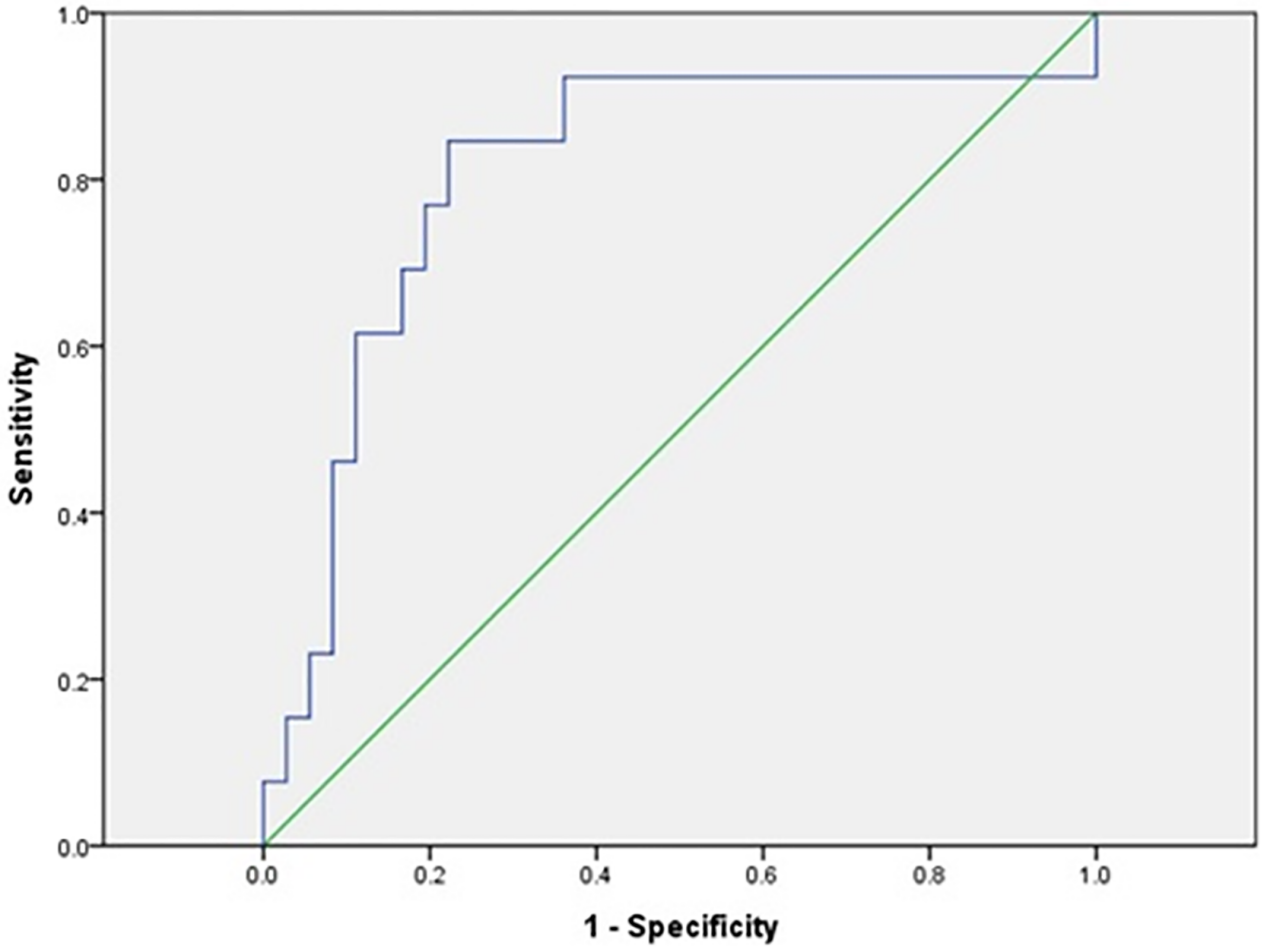
Combined receiver operating characteristic curve of the % change of SPP during CPB for the postoperative 6 h hyperlactatemia (peak lactate > 3mmol/L). An area of 0.808 (95% confidence interval of 0.652–0.963, P = 0.001) was observed below the line of % change in SPP value. The optimal cutoff value for postoperative 6 h hyperlactatemia was 48% decrease from the baseline SPP (after induction of anesthesia), with a sensitivity and specificity of 84.6% and 77.8%, respectively. SPP = skin perfusion pressure; CPB = cardiopulmonary bypass.

Considering its association with postoperative 6 h peak lactate level, the SPP value during CPB was further analyzed with the changes from baseline. In the ROC curve analysis of the % change in SPP during CPB for discriminating between patients with and without postoperative 6 h hyperlactatemia, an AUC of 0.808 (95% CI,0.652–0.963, P = 0.001) was observed. The optimal cutoff value was 48% decrease with a sensitivity and specificity of 84.6 and 77.8%, respectively (Fig 2).

Comparisons of perioperative data between patients whose SPP decreased >48% (large drop, n = 20) and those whose SPP decreased ≤48% or increased (no large drop, n = 51) are shown in Table 3. Preoperative data and operative details including the pRBC transfusion and use of milrinone and vasopressors were similar between the groups. Postoperatively, incidences of AKI (6/20 vs. 1/51, P = 0.002), mechanical ventilation >24 h (P = 0.020), and a composite endpoint of 30-day morbidity (3/20 vs. 0, P = 0.003) were significantly higher in the large drop group than in the no large drop group.

**Table 3:** Perioperative data according to the % change of SPP during CPB from the baseline value.

	Large drop group > 48% (n = 20)	No large drop group ≤ 48% or increased (n = 51)	P value
Age, yr	64 ± 9	58 ± 12	0.065
Female sex	11 (55)	25 (49)	0.650
Body surface area, m^2^	1.57 [1.48−1.78]	1.64 [1.52−1.79]	0.961
Hypertension	7 (35)	24 (47)	0.357
Diabetes	1 (5)	4 (8)	1.000
Chronic obstructive pulmonary disease	0	2 (4)	1.000
EuroSCORE	3 [2–4]	3 [2–5]	0.737
Left ventricular ejection fraction, %	69 ± 7	64 ± 11	0.069
New York Heart Association class 3, 4	7 (35)	7 (15)	0.099
Medications			
Beta blockers	3 (15)	14 (28)	0.361
Calcium channel blockers	5 (25)	10 (20)	0.748
Renin angiotensin system blockers	6 (30)	18 (35)	0.671
Previous cardiac surgery	5 (25)	10 (20)	0.748
Preoperative hemoglobin, mg/dL	11.5 [10.3–12.5]	12.3 [11.2–13.3]	0.209
Preoperative creatinine, mg/dL	0.87 [0.64–0.96]	0.78 [0.64–0.97]	0.052
Preoperative lactate, mmol/L	1.0 [0.8–1.3]	1.0 [0.9–1.3]	0.166
Intraoperative data			
Aortic valve surgery ± aorta procedure	4 (20)	11 (22)	0.884
Mitral valve surgery ± TAP or MAZE	10 (50)	29 (57)	0.601
Double valve surgery	6 (22)	11 (22)	0.540
Cardiopulmonary bypass time, min	121 [97–168]	115 [88–150]	0.985
Aortic cross clamping time, min	87 [58–125]	85 [59–121]	0.862
pRBC transfusion	10 (50)	19 (37)	0.326
Amount of norepinephrine, μg	58.0 [28.0–94.0]	44.4 [30.0–77.4]	0.089
Patients received milrinone	4 (20)	10 (20)	1.000
Postoperative 6 h cardiovascular drugs			
Patients received vasopressors	9 (45)	28 (56)	0.405
Patients received milrinone	11 (46)	12 (26)	0.084
Postoperative 6 h pRBC transfusion	10 (50)	21 (41)	0.500
Length of intensive care unit stay, days	2 [1–4]	2 [1–3]	0.283
Length of hospital stay, days	11 [9–23]	10 [8–13]	0.182
Thirty day morbidity endpoints			
Stroke	2 (10)	0	0.076
Myocardial infarction	0	2 (4)	1.000
Acute kidney injury	6 (30)	1 (2)	0.002
Mechanical ventilation > 24 h	3 (15)	0	0.020
Deep sternal wound infection	0	0	
Reoperation	2 (11)	1 (2)	0.177
Mortality	0	0	
Composite	8 (40)	4 (8)	0.003

Values are mean ± SD, median [IQR], or n (%). SPP = skin perfusion pressure; CPB = cardiopulmonary bypass; TAP = tricuspid annuloplasty; pRBC = packed red blood cells.

In the univariate analysis for a composite endpoint of 30-day morbidity, sex, NYHA class≥3, CRSO_2_ after induction of anesthesia, large drop group, CRSO_2_ after weaning from CPB, and postoperative 6 h hyperlactatemia showed statistical significance. In the multivariate model, only the large drop group was identified as an independent predictor of a composite endpoint of 30-day morbidity (OR, 12.84; 95% CI, 1.48–111.42; P = 0.021) (Table 4).

**Table 4:** Predictive power of selective variables for composite endpoint of 30-day morbidity according to logistic regression analysis.

Variables	Univariate		Multivariate	
	OR (95% CI)	P value	OR (95% CI)	P value
Age	1.07 (1.00–1.14)	0.061		
Female sex	6.35 (1.28–31.52)	0.024	10.80 (0.78–149.61)	0.076
Chronic kidney disease	7. 00 (0.86–56.90)	0.069		
NYHA class ≥3	8.17 (1.99–30.55)	0.003	3.88 (0.40–37.24)	0.240
CRSO_2_ at Post-IND	0.91 (0.85–0.98)	0.016	0.98 (0.87–1.10)	0.717
Large drop group (CPB-SPP)	16.33 (3.07–87.04)	0.001	12.84 (1.48–111.42)	0.021
CRSO_2_ after CPB-off	0.93 (0.87–0.99)	0.017	0.98 (0.87–1.10)	0.715
PADP postoperative 6 h	1.15 (0.87–1.35)	0.079		
Postop 6 h hyperlactatemia	3.68 (1.57–8.68)	0.003	6.89 (0.88–53.79)	0.066

Large drop group (CPB-SPP) = Patients whose SPP during CPB were decreased >48% from the baseline value; NYHA = New York Heart Association; CRSO_2_ = cerebral regional oxygen saturation; Post-IND = after induction of anesthesia; SPP = skin perfusion pressure; CPB = cardiopulmonary bypass; PADP = pulmonary arterial diastolic pressure; Postop 6 h hyperlactatemia = peak lactate level > 3mmol/L; OR = odds ratio; CI = confidence interval.

## Discussion

In this prospective observational study, we observed a potentially promising role of SPP as an indicator of tissue perfusion in patients undergoing valvular heart surgery. A significant negative correlation between SPP value during CPB and postoperative 6 h peak lactate level was observed. Decrease in SPP of >48% during CPB from the baseline value could identify patients at risk of developing postoperative 6 h hyperlactatemia and was associated with 12.8 times higher risk of a composite endpoint of postoperative 30-day morbidity.

Current literatures suggest that targeting normalization of systemic hemodynamic parameters, which constitute the main resuscitation goals in critically ill patients, would not ensure improved organ perfusion and clinical outcomes [3–5, 7]. Consistently, no independent association was observed between most of these variables and postoperative hyperlactatemia or morbidity in the current study. This may verify that optimal tissue perfusion cannot be appropriately guided by conventional hemodynamic monitoring in cardiac surgery with CPB. Notably, microcirculatory disturbances, which was related to delayed lactate clearance or hyperlactatemia, could be detected using the NIRS-tissue RSO_2_ monitoring or sublingual video-microscopy in cardiac surgical patients [23, 24]. However, these devices lack consistency regarding their usefulness to predict clinical deterioration associated with tissue hypoperfusion during CPB [23–25]. Undoubtedly, the risk of compromised microcirculation is highest during CPB throughout the perioperative period. In this study, as presumed, a large decrease in SPP during CPB could discriminate whether early postoperative hyperlactatemia as well as 30-day morbidity would occur.

SPP, which is widely used for noninvasive assessment of microcirculation of the lower extremities in patients with PAD, diabetes, or renal failure, has been validated for its accuracy and reliability [11–13]. Particularly in patients with critical limb ischemia, the SPP has shown its superiority over other methodologies, including even the angiographic findings, in assessing perfusion restoration or predicting long-term prognosis associated with tissue viability [14, 15]. It reflects a post-occlusive reactive hyperemia and, thus, addresses the functional aspects of microvasculature [12, 15], which is in contrast to conventional indicators of peripheral perfusion such as skin temperature or CPTG that primarily reflect the vascular tone only [26]. The recovery slope following vascular occlusion test (VOT) with thenar NIRS, which also indicates microvascular reactivity, provided more accurate information regarding perfusion abnormalities and subsequent organ failure in human sepsis compared with StO_2_ alone [27]. A pilot study of the measurement of VOT recovery slope in cardiac surgery revealed a decline by 40% during CPB, whereas the StO_2_ was not changed from the baseline level; however, its association with the established endpoints of tissue hypoperfusion or clinical deterioration was not further determined [28]. Similarly, the SPP value was decreased by an average of 30% in our study participants, and nearly one third of them whose SPP dropped >48% was closely associated with postoperative hyperlactatemia. Furthermore, such a reduction in SPP was significantly linked to increased postoperative morbidities, particularly AKI. Even in the absence of an overt surgically induced ischemia-reperfusion injury, the renal medulla is particularly vulnerable to injury from microcirculatory disturbances as it already receives hypoxic blood due to its unique vascularity, which explains the frequent development of AKI after CPB [21, 29]. In line with the results of our study, a previous retrospective study reported a strong association between lower VOT recovery slope at postoperative day 1 and in-hospital complications, including severe renal failure following cardiac surgery [30], although the tests were performed only at the pre- and postoperative period without intraoperative analysis. Of note, patients’ characteristics or operative data showed no significant differences between patients who exhibited a drop in SPP >48% or not, making it difficult to anticipate beforehand for preoperative risk stratification, as well as limiting subsequent researches aimed at elucidating preventive measures.

The regional hypoperfusion induced by CPB is known to result in postoperative organ dysfunction [31]. Loss of coherence between the macro- and microcirculation may be inevitable during CPB with major inflammatory insult, severe hemodilution, and extreme changes in temperature, although clinicians can control systemic blood flow almost completely during CPB [32]. Nevertheless, no hemodynamic target ensuring optimal perfusion that gained wide clinical acceptance exists, and the majority of conventional hemodynamic indices may be expressed as distorted or even unavailable during CPB [31]. Serum lactate concentration also has an inevitable drawback to be used as a guidance of perfusion during CPB, such as a time-lag [9]. In that context, the result of the current study provide a clinically valuable suggestion that the microvascular reactivity represented by the SPP value might be an appropriate target reflecting optimal perfusion during CPB. With particular relevance to renal protection following cardiac surgery, which greatly depends on the adequacy of perfusion during CPB [29], SPP monitoring has a potentially meaningful role that merits to be proven.

Norepinephrine and milrinone administered during study period could have affected SPP values in the current study, although it is not clear whether these agents would augment or disrupt peripheral perfusion. In this context, there is an ongoing debate over the impact of vasoactive agents on microcirculation [33–35]. Similarly, transfusion of RBCs might have influenced on SPP, lactate, and even the clinical outcomes. Despite that there were no significant differences in comparison of these factors between the Large drop group and No large drop group, we have additionally analyzed with adjustments by propensity score based on transfusion, amount of norepinephrine, and use of milrinone to achieve their optimal balance. In that additional analysis, we still could observe a statistically significant predictability of large drop of SPP during CPB on 30-day morbidity (odds ratio 5.981 with 95% confidence interval 1.151–31.083), which may more firmly support its significance in risk stratification.

The limitations of our study are as follows: First, potential non-hypoxic causes of hyperlactatemia could not be completely excluded, which could have led to some possible misinterpretation of the current result. Nonetheless, its clinical importance is now beyond the debates over multifactorial origins. Even the relatively mild elevation in lactate level shortly after surgery may provide early estimates of the significant clinical deterioration in the current era of advanced patient care [36]. Second, unlike the critical values which are available for PAD patients [11, 12, 15], the cutoff point of the actual SPP value that can simply categorize patients into the high- or low-risk group was not identified in the current analysis. Third, although we did not observe significant differences in other patient and operative data besides vasoactive agents and transfusion between the Large drop group and the other group, possible confounding effects of critical influential factors, such as preoperative cardiovascular performance or temperature of SPP measuring site, might not have been completely ruled out. Moreover, the sample size may have been small to determine the direct relationship between the absolute SPP value and the clinical outcomes. Further validation on a large scale study will be required to resolve these matters.

## Conclusions

In summary, we firstly provide primary evidence regarding the utility of SPP monitoring as an early indicator of tissue perfusion in cardiac surgery using CPB. Decrease in SPP value of >48% during CPB from the baseline could predict postoperative 6 h hyperlactatemia as well as a composite of 30-day morbidity. SPP monitoring during CPB could be a potentially promising method to be targeted in future hemodynamic management aimed at improving tissue oxygenation and prognosis in cardiac surgery.

## Supporting information

S1 Fig Combined receiver operating characteristic curve for the perioperative peak serum lactate to discriminate a composite endpoint of 30-day morbidity. Areas of 0.762 (95% confidence interval of 0.602–0.922, P = 0.004) and 0.701 (95% confidence interval of 0.550–0.852, P = 0.029) were observed below the line of postoperative 6 h and 12 h peak lactate level, respectively. The optimal cutoff value for predicting composite endpoint was 3.0 mmol/L of postoperative 6 h peak lactate with a sensitivity and specificity of 66.7% and 84.7%, respectively.
(TIF)

S1 File Study protocol approved by ethics committee. (DOC)

S2 File STROBE statement. (DOC)

S3 File Raw data. (XLSX)
