# Supplementary figures and images for: Skin perfusion pressure as an indicator of tissue perfusion in valvular heart surgery: Preliminary results from a prospective, observational study
Source: PLoS One. 2017 Sep 19;12(9):e0184555. doi: 10.1371/journal.pone.0184555 (PMC5604958; doi:10.1371/journal.pone.0184555)

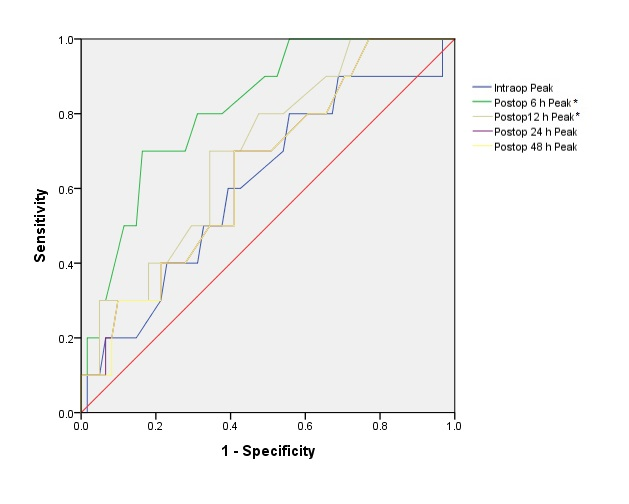

Supplement: S1 Fig — Areas of 0.762 (95% confidence interval of 0.602–0.922, P = 0.004) and 0.701 (95% confidence interval of 0.550–0.852, P = 0.029) were observed below the line of postoperative 6 h and 12 h peak lactate level, respectively. The optimal cutoff value for predicting composite endpoint was 3.0 mmol/L of postoperative 6 h peak lactate with a sensitivity and specificity of 66.7% and 84.7%, respectively. (TIF) [file pone.0184555.s001.tif]
