# Supplementary material for: Skin perfusion pressure as an indicator of tissue perfusion in valvular heart surgery: Preliminary results from a prospective, observational study
Source: PLoS One. 2017 Sep 19;12(9):e0184555. doi: 10.1371/journal.pone.0184555 (PMC5604958; doi:10.1371/journal.pone.0184555)
Supplement: S1 File — (DOC) [file pone.0184555.s002.doc]

**Clinical Study Protocol**

(Version 1.0)

1. **Study Title**

Korean: 체외순환을 사용하는 심장 수술을 시행 받는 환자에서 조직의 산소화 지표로서 skin perfusion pressure 감시의 유용성

English: Assessment of skin perfusion pressure as an indicator of tissue oxygenation in cardiac surgery patients undergoing cardiopulmonary bypass

Investigators

Principal Investigator: Young Song

Co-Investigators: Young-Lan Kwak, Seung Hyeon Lee, Bora Lee

**2. Necessity of Study and Overview**

**1) Current statues of perioperative hemodynamic monitoring in cardiac surgery**

Cardiac surgery is characterized by organ damage and consequent complications caused by pre-existing cardiac disease, cardiopulmonary bypass (CPB), and surgical insult (1). Adequate oxygen delivery is prerequisite to reduce organ dysfunction (2), which requires hemodynamic stability. Conventional perioperative hemodynamic monitor comprises of systemic arterial pressure derived from radial and femoral artery, cardiac index (CI) and mixed venous oxygen saturation (SvO2) derived from pulmonary artery catheterization, and cerebral oxygen saturation (rSO2) with the use of near infrared spectroscopy (3). But these are reported to be unable to reflect microcirculation (4-7).

**2) Implication of peripheral perfusion monitoring and previous studies**

Microcirculation is recognized as an end organ playing an important role for a pathophysiology in critical care and cardiac surgical cohort (8). In sepsis or ischemia reperfusion injury, heterogeneity in tissue perfusion increases and oxygen extraction property decreases, which result in organ damage (9-12). Moreover, peripheral perfusion and oxygen delivery are impaired in hemodynamically unstable states because distribution of blood flow is concentrated to vital organs (7, 13). The main purpose of hemodynamic monitoring in critical care room and operating theater is to prevent progression to organ failure by improving the tissue oxygenation when insufficient. Hence, apart from the major organs, the importance of indicators to monitor peripheral circulation that early perfusion drops occurs is increasingly emphasized (14). Tissue oxygen saturation (StO2) is a widely known noninvasive device, which is usually monitored at thenar muscle using the near infrared spectroscopy (15-17). However, it is interfered with other pigment molecules such as skin deposits and cannot be distinguished from hemoglobin and myoglobin. The high cost and time consuming nature of it also makes it difficult to commercialize (22).

1. **Skin perfusion pressure (SPP), its possible role as a perioperative monitoring device**

Skin perfusion pressure (SPP) is an index encompassing the functional aspects of microcirculation because it uses the principle of measurement of blood flow after occlusion (23). The laser Doppler sensor senses the red blood cells themselves rather than the pigment molecules in the restoration of blood flow after occlusion (Figure 1), which enable the SPP more directly reflects tissue perfusion compared to StO2.


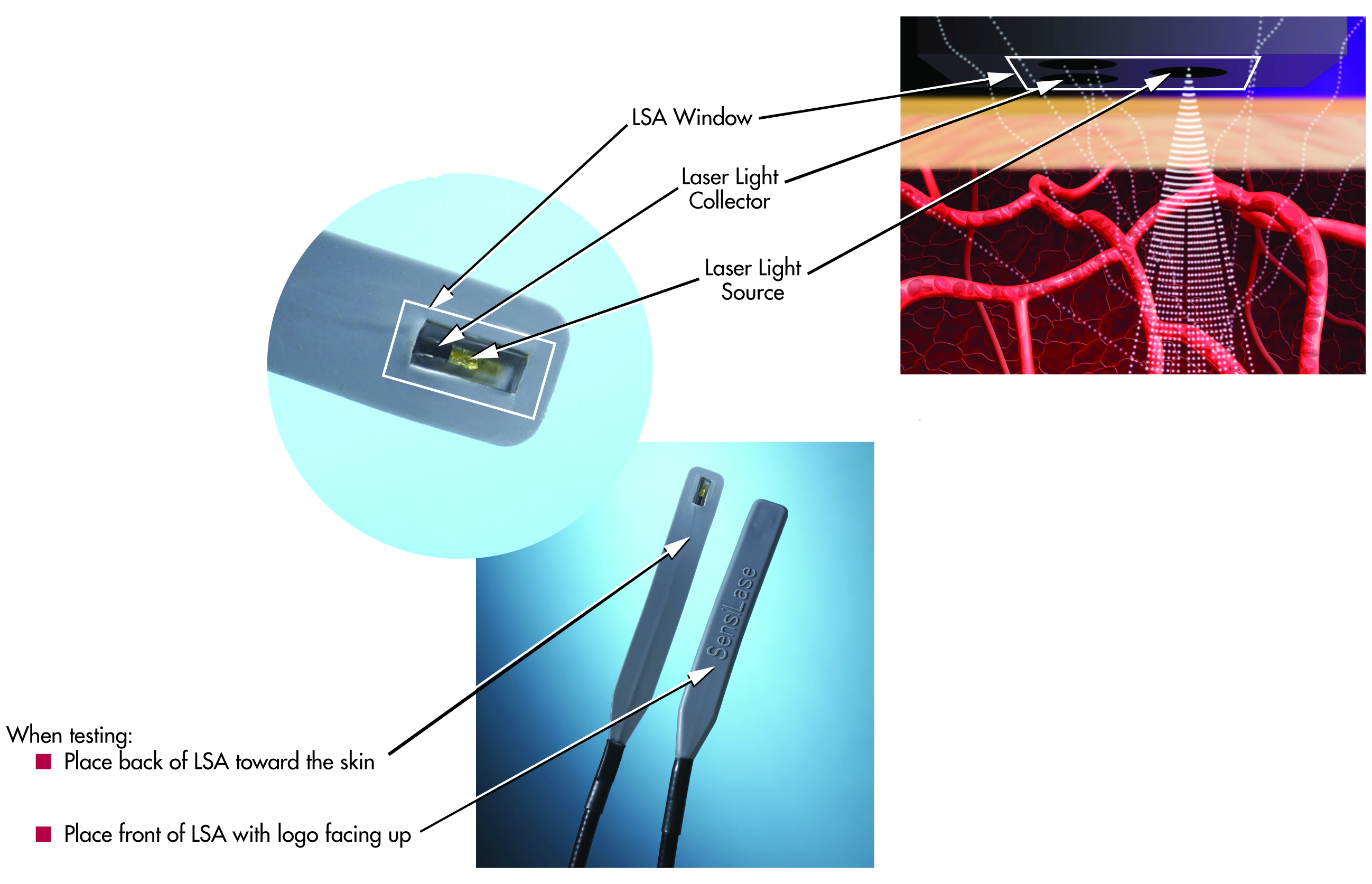


**Figure 1. Laser sensor detects Doppler shift by red blood cells occurs in blood flow restoration**

The measure is performed automatically. The machine is operated after the cuff is wound on the foot and the pressure is increased until the capillary blood flow disappears and after then blood flow is resumed while the laser sensor attached under the cuff detects it (Figure 2). In healthy adults, lower extremity SPP was reported to be about 10 mmHg lower than systemic mean arterial pressure [24]. In addition, studies on peripheral vascular disease have reported that the SPP value measured at the foot > 50 mmHg may reflects "adequate perfusion" [25-27].


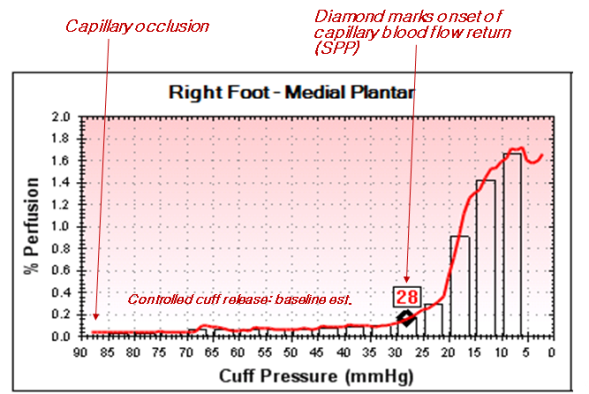


Figure 2. After capillary occlusion by raising pressure, the cuff is slowly deflated and the laser Doppler sensor continuously monitors blood flow resumption and records the pressure at which blood flow begins to appear in the capillary (Black Diamond). In this figure, the SPP is 28 mmHg.

SPP has been proven to be useful in diagnosing critical limb ischemia, identifying treatment effects after intervention, determining the level of limb amputation, and anticipation for ulcer healing [28-30]. However, its role as a monitoring tool in other clinical circumstances where microcirculatory disturbance may occur has never been evaluated.

**3. Study Objectives**

To investigate whether SPP can be used as an indicator of microcirculation during and after weaning off CPB in patients undergoing cardiac surgery.

Primary objective: To determine relationship between SPP values assessed during operation and postoperative 6 h and peak serum lactate level during postoperative 48 h

Secondary objective: To determine ability of SPP to predict postoperative hyperlactatemia and major morbidity

**4. Study Site and Duration**

Study Site: Yonsei University Health System, Severance Hospital, Seoul, Republic of Korea

Study Duration: 12 months after the approval of IRB

**5. Inclusion/Exclusion Criteria and Screening Items**

1) Inclusion criteria: A patient at the age ≥ 20 years old who will undergo the elective on-pump valvular surgery

2) Exclusion criteria:

known peripheral arterial occlusive disease, patients without swan ganz catheter insertion, heart transplantation, infective endocarditis, hemodynamic instability requiring vasopressor, preoperative lactate > 2mmol/L, known liver cirrhosis, pregnant, cognitive dysfunction, being unable to communicate

3) Screening Items: Medical history, primary carcinoma, dosage and period of preoperative chemotherapy medication regimen, routine chemistry assay, hematologic assay, urinalysis, chest X-ray, ECG.

**6. Target number of subjects and the calculation basis**

Correlation coefficient between the average of intraoperative rSO2 value and postoperative 4 h peak lactate level who underwent valvular heart surgery in our institution before was 0.20. A previous report demonstrated 0.46 of correlation coefficient between thenar muscle StO2 and serum lactate clearance in cardiac surgery [31-> 논문17]. Assuming a similar relationship between SPP and lactate level, the number required to detect a difference of 0.25 between correlation coefficients of rSO2-lactate and SPP-lactate with the power ≥ 80% at the significance level of 5% is 66 subjects. Considering the drop-out rate as 10%, the study is to be implemented in 72 patients per.

(Analysis Tool: PASS version (NCSS Statistical Software, 2013).

**7. Study Design and Methods**

1) Enrollment

The researcher will determine whether to enroll the patient who meets the inclusion criteria among those who are decided to be performed with surgery, and explain the purpose and methods of the study to the patient and his/her legally acceptable representative guardian at a treatment room of the ward that is an independent space. One hour later he will re-visit the treatment room to obtain consent. If a patient who has agreed to participate in the research is screened and satisfies the selection criteria, he/she is finally enrolled in the study.

Before inducing anesthesia, after re-confirming on the patient’s consent (continuous verification),

2) Perioperative management

Anesthesia management, operation method, and postoperative management except SPP measurement are performed according to our institutional standard. After the patient is lying on the surgical bed, the INVOSTM Somasensor is attached to both sides of the forehead and connected to the INVOS 5100C (Covidien, Boulder, CO, USA) to begin monitoring the rSO2. Before the induction of anesthesia, radial artery catheter insertion for continuous blood pressure measurement is performed under local anesthesia. Anesthesia was induced with midazolam (0.03-0.05 mg / kg) and sufentanil (1.5-2 ㎍ / kg), followed by sufentanil (0.2-0.3 ㎍ / kg / h) continuous sedation and sevoflurane 0.6-2.0%. After the induction of anesthesia, a pulmonary artery catheter (Swan-Ganz CCombo CCO / SvO2®, Edwards Lifesciences LLC, USA), which can measure CI and SvO2, is mounted through the right jugular vein and measurement of these indicators is started. CPB is performed by inserting one or two venous catheters into the ascending aorta following the median sternotomy and using a non-invasive pump (Edwards Vital ™, Eswards Lifesciences, USA), maintaining homogeneous perfusion at 2.0-2.4 L / m2 / min. The pump circuit is filled with 100 mL of 20% albumin, 0.5 g/kg of 20% mannitol, sodium bicarbonate (20 mEq), heparin 2000 IU, and 1000 mL acetate rinse solution. During the CPB, myocardial protection is controlled by controlling the body temperature with a mild hypothermia (nasopharyngeal 32-33C).

After the CPB was completed, the patient was given 4-6 mL / kg / h of crystalloid and colloid is administered to compensate blood loss up to 20 mL/kg per day maximally. During CPB, the hematocrit is maintained at 20% or more (hemofiltration if necessary) and remains above 25% before and after CPB. The management of fluid and blood transfusion in the ICU is performed according to the standard protocol, taking into account the pulmonary status, urine volume, and hemodynamic parameters. The mean arterial pressure is maintained at 60-80 mmHg with the support of norepinephrine (max. 0.3 μg / kg / min) and vasopressin. If CI is less than 2.0 L / min / m2 despite adequate intravascular volume, milrinone is given.

3) Study protocol

SPP was measured at the dorsum of both foot using SensiLase PAD-IQ (Vasamed Inc., Eden Prairie, MN, USA). Measurements are made in the dorsum area, which is less likely to have an undiagnosed peripheral artery occlusion (Fig. 3). We will attach the laser Doppler sensor to the area where the beating of the dorsalis pedis is felt on both feet, and wrap the 8 cm wide cuff over it. When the pressure of the cuff increases and the perfusion value of the capillary artery of the affected part becomes less than 0.1 volume%, the pressure gradually decreases to 5 mmHg and the blood flow resumes. The SPP (mmHg) is the cuff pressure at the time when the laser Doppler resumes blood flow for the first time and is automatically recorded on the main body screen (Fig. 4). Immediately after induction of anesthesia, measure the SPP and reattach the sensor and cuff if necessary.


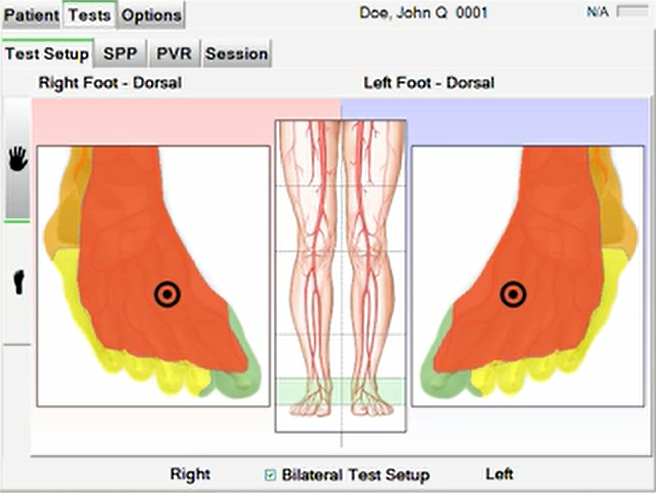

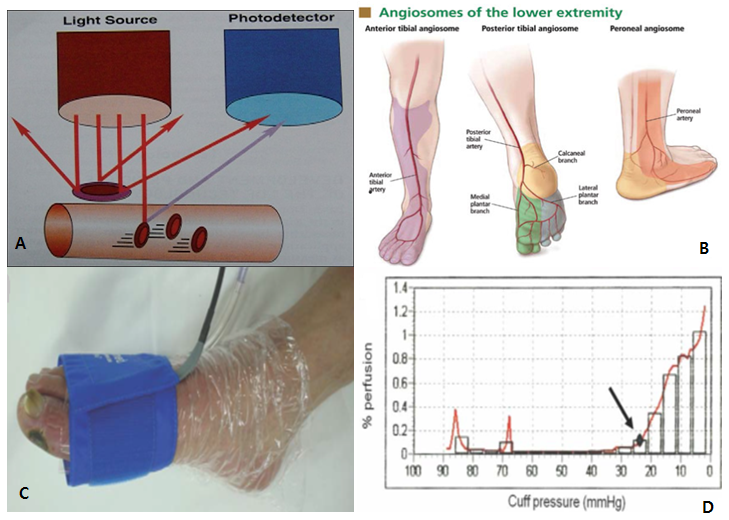


Figure 3. Selection of vascular distribution area

Figure 4. The SPP value is displayed on the screen.

**8. Clinical Examination Items and Observational Examination Methods**

1) Pulse rate, radial artery pressure, central venous pressure and pulmonary artery pressure, CI, SvO2, central-peripheral body temperature (nasopharynx and skin), SPP

Time point: after induction of anesthesia, during hypothermic CPB repeated at 30-min intervals, after weaning from CPB, after sternal closure, after arrival at the ICU, and postoperative 0,2,4,6 h

2) Arterial blood lactate - assessment time point: after induction of anesthesia, during hypothermic CPB repeated at 30-min intervals, after weaning from CPB, after sternal closure, after arrival at the ICU, and postoperative 0,2,4,6, 12, 24, and 48 h [32].

3) Patient demographic data, Intraoperative data

4) Postoperative data: requirement of vasopressor and inotropic agents, pRBC transfusion, and 30-day morbidity endpoints, including new-onset myocardial infarction, acute kidney injury, stroke, mechanical ventilation >24 h, deep sternal wound infection, reoperation, mortality, and composite of the above.

** The confidentiality of the study data should be kept by recording the patient using the serial number in the CRF.

**9. Expected Adverse Events and Precautions for Use**

Sensilase® PAD-IQ ™ (Vasamed, Eden Prairie, MN, USA) is approved by the Korean Food and Drug Administration. And it has been widely used in patients undergoing interventional procedures for peripheral arterial occlusive disease since May 2014 in our institution. The occurrence of side effects and complications from SPP measurements has not been reported in this center or other centers. There are no known adverse effects, but cuff and sensors may cause skin contact sensitization. In addition, signals may be subject to interference with the use of electrocautery during surgery and the intense movement of the measurement site. Hence, measurement will be under control these issues.

**10. Safety Assessment Method including Adverse Events and Response Measures against Adverse Events**

There are no known adverse effects but SPP we will check the foot skin after the first measure of SPP and after the surgery to see if erosion or redness has occurred in every participant.

**11. Criteria for Discontinuation of the Clinical Study and Drop-out**

If erosion, redness, or other contact hypersensitivity occurs.

**10. Statistical Analysis Method**

For the continuous variables, the regular distribution is to be confirmed by a normality test. Parametric data will be compared between the groups by using an independent t-test, whereas non-parametric data will be compared between the groups by using Mann-Whitney U test. And the results will be indicated by using the mean ± standard deviation or the median value ± inter-quartile displacement (1 quartile - 3 quartile). For discrete variables, Chi-Square test or Fisher’s exact test will be conducted and the result is to be indicated by the number of patients (percentage). When p value is <0.05, it will be considered as statistically significant. For statistical analysis, Statistical Package for Social Sciences statistical software (SPSS 20.0, USA) and SAS software 9.2 (SAS Inc., Cary, NC, USA) will be used.

**12. Plan for report of harmful cases**

Reports of serious adverse events (problems with device-contacted skin) and unexpected problems and research-related adverse events are reported to the IRB within 7 days, and within 24 hours for serious and unexpected adverse events.

**13. Data Safety Monitoring Plan (**DSMP**)**

Professor Young Song will make comparisons between the source documents and the study protocol at every 6 months to assure the completeness of data and will review the safety data of subjects. The subject identification code on the data will be encoded, and will be filed in a safe installed with a locking device while the electronic document will be saved in a computer which is restricted for access.

**14. Medical Device Management Plan**

The machine management site is the cardiovascular hospital hybrid operating room, and the manager is the cardiovascular hospital operating room nursing nurse.

**15. Statistical analysis**

The correlation between the SPP and the highest lactate within 48 hours postoperatively will be assessed using Pearson's correlation test. For the comparison of continuous variables, independent t-test or Mann-Whitney U test was used. Categorical variables were compared using the chi-square or Fisher’s exact test, as appropriate. The SPP's predictive power for hyperlactatemia and major complication will be determined by area under receiver operator characteristic Curve and obtain the optimum cut-off value. P <0.05 is statistically significant.

**16. Timetable**

- Study Implementation: After approval of IRB ~ 12^th^ months

- Statistic Processing of the Study Data and Analysis of the Results: 13^th^ months

- The Clinical Study Report and Preparation of Study Paper: 14^th^ months

**17. References**

1. Westaby S. Organ dysfunction after cardiopulmonary bypass. A systemic inflammatory reaction initiated by the extracorporeal circuit. *Intensive care medicine* 1987; **13**(2): 89-95.

2. Shoemaker WC, Appel PL, Kram HB. Tissue oxygen debt as a determinant of lethal and nonlethal postoperative organ failure. *Critical care medicine* 1988; **16**(11): 1117-20.

3. Cannesson M, Pestel G, Ricks C, Hoeft A, Perel A. Hemodynamic monitoring and management in patients undergoing high risk surgery: a survey among North American and European anesthesiologists. *Critical care* 2011; **15**(4): R197.

4. Zanotti Cavazzoni SL, Dellinger RP. Hemodynamic optimization of sepsis-induced tissue hypoperfusion. *Critical care* 2006; **10 Suppl 3**: S2.

5. De Backer D, Creteur J, Preiser JC, Dubois MJ, Vincent JL. Microvascular blood flow is altered in patients with sepsis. *American journal of respiratory and critical care medicine* 2002; **166**(1): 98-104.

6. Rady MY, Rivers EP, Nowak RM. Resuscitation of the critically ill in the ED: Responses of blood pressure, heart rate, shock index, central venous oxygen saturation, and lactate. *Am J Emerg Med* 1996; **14**(2): 218-25.

7. Marik PE, Levitov A, Young A, Andrews L. The Use of Bioreactance and Carotid Doppler to Determine Volume Responsiveness and Blood Flow Redistribution Following Passive Leg Raising in Hemodynamically Unstable Patients. *Chest* 2013; **143**(2): 364-70.

8. Koning NJ, Vonk ABA, van Barneveld LJ, et al. Pulsatile flow during cardiopulmonary bypass preserves postoperative microcirculatory perfusion irrespective of systemic hemodynamics. *J Appl Physiol* 2012; **112**(10): 1727-34.

9. Boerma EC, Mathura KR, van der Voort PHJ, Spronk PE, Ince C. Quantifying bedside-derived imaging of microcirculatory abnormalities in septic patients: a prospective validation study. *Critical care* 2005; **9**(6): R601-R6.

10. De Backer D, Dubois MJ, Schmartz D, et al. Microcirculatory Alterations in Cardiac Surgery: Effects of Cardiopulmonary Bypass and Anesthesia. *Ann Thorac Surg* 2009; **88**(5): 1396-403.

11. Boston US, Slater AM, Orszulak TA, Cook DJ. Hierarchy of regional oxygen delivery during cardiopulmonary bypass. *Ann Thorac Surg* 2001; **71**(1): 260-4.

12. Mollnes TE, Fiane AE. Distribution and hierarchy of regional blood flow during hypothermic cardiopulmonary bypass - Invited commentary. *Ann Thorac Surg* 2001; **72**(2): 547-.

13. Sullivan MJ, Knight JD, Higginbotham MB, Cobb FR. Relation between Central and Peripheral Hemodynamics during Exercise in Patients with Chronic Heart-Failure - Muscle Blood-Flow Is Reduced with Maintenance of Arterial Perfusion-Pressure. *Circulation* 1989; **80**(4): 769-81.

14. Lima A, Bakker J. Noninvasive monitoring of peripheral perfusion. *Intensive care medicine* 2005; **31**(10): 1316-26.

15. Scheeren TW, Schober P, Schwarte LA. Monitoring tissue oxygenation by near infrared spectroscopy (NIRS): background and current applications. *Journal of clinical monitoring and computing* 2012; **26**(4): 279-87.

16. Deblasi RA, Ferrari M, Natali A, Conti G, Mega A, Gasparetto A. Noninvasive Measurement of Forearm Blood-Flow and Oxygen-Consumption by near-Infrared Spectroscopy. *J Appl Physiol* 1994; **76**(3): 1388-93.

17. Lima A, van Bommel J, Sikorska K, et al. The relation of near-infrared spectroscopy with changes in peripheral circulation in critically ill patients. *Critical care medicine* 2011; **39**(7): 1649-54.

18. Gomez H, Torres A, Polanco P, et al. Use of non-invasive NIRS during a vascular occlusion test to assess dynamic tissue O-2 saturation response. *Intensive care medicine* 2008; **34**(9): 1600-7.

19. Dakak N, Husain S, Mulcahy D, et al. Contribution of nitric oxide to reactive hyperemia - Impact of endothelial dysfunction. *Hypertension* 1998; **32**(1): 9-15.

20. Celermajer DS, Sorensen KE, Gooch VM, et al. Non-invasive detection of endothelial dysfunction in children and adults at risk of atherosclerosis. *Lancet* 1992; **340**(8828): 1111-5.

21. Binggeli C, Spieker LE, Corti R, et al. Statins enhance postischemic hyperemia in the skin circulation of hypercholesterolemic patients - A monitoring test of endothelial dysfunction for clinical practice? *J Am Coll Cardiol* 2003; **42**(1): 71-7.

22. Myers D, McGraw M, George M, Mulier K, Beilman G. Tissue hemoglobin index: a non-invasive optical measure of total tissue hemoglobin. *Critical care* 2009; **13**.

23. Obeid AN, Barnett NJ, Dougherty G, Ward G. A Critical-Review of Laser Doppler Flowmetry. *J Med Eng Technol* 1990; **14**(5): 178-81.

24. Holstein P, Lund P, Larsen B, Schomacker T. Skin perfusion pressure measured as the external pressure required to stop isotope washout. Methodological considerations and normal values on the legs. *Scandinavian journal of clinical and laboratory investigation* 1977; **37**(7): 649-59.

25. Yamada T, Ohta T, Ishibashi H, et al. Clinical reliability and utility of skin perfusion pressure measurement in ischemic limbs--comparison with other noninvasive diagnostic methods. *Journal of vascular surgery* 2008; **47**(2): 318-23.

26. Castronuovo JJ, Jr., Adera HM, Smiell JM, Price RM. Skin perfusion pressure measurement is valuable in the diagnosis of critical limb ischemia. *Journal of vascular surgery* 1997; **26**(4): 629-37.

27. Tsai FW, Tulsyan N, Jones DN, Abdel-Al N, Castronuovo JJ, Jr., Carter SA. Skin perfusion pressure of the foot is a good substitute for toe pressure in the assessment of limb ischemia. *Journal of vascular surgery* 2000; **32**(1): 32-6.

28. Castronuovo JJ, Adera HM, Smiell JM, Price RM. Skin perfusion pressure measurement is valuable in the diagnosis of critical limb ischemia. *Journal of vascular surgery* 1997; **26**(4): 629-37.

29. Urabe G, Yamamoto K, Onozuka A, Miyata T, Nagawa H. Skin Perfusion Pressure is a Useful Tool for Evaluating Outcome of Ischemic Foot Ulcers with Conservative Therapy. *Annals of vascular diseases* 2009; **2**(1): 21-6.

30. Adera HM, James K, Castronuovo JJ, Jr., Byrne M, Deshmukh R, Lohr J. Prediction of amputation wound healing with skin perfusion pressure. *Journal of vascular surgery* 1995; **21**(5): 823-8; discussion 8-9.

31. Mozina H, Podbregar M. Near-infrared spectroscopy during stagnant ischemia estimates central venous oxygen saturation and mixed venous oxygen saturation discrepancy in patients with severe left heart failure and additional sepsis/septic shock. *Critical care* 2010; **14**(2): R42.

32. Ranucci M, De Toffol B, Isgro G, Romitti F, Conti D, Vicentini M. Hyperlactatemia during cardiopulmonary bypass: determinants and impact on postoperative outcome. *Critical care* 2006; **10**(6): R167.
